# Dementia and traumatic brain injuries: underestimated bidirectional disorder

**DOI:** 10.3389/fneur.2023.1340709

**Published:** 2024-01-08

**Authors:** Ayman El-Menyar, Hassan Al-Thani, Mohamed Farouk Mansour

**Affiliations:** ^1^Clinical Research, Trauma and Vascular Surgery, Hamad Medical Corporation, Doha, Qatar; ^2^Weill Cornell Medical School, Doha, Qatar; ^3^Trauma and Vascular Surgery, Hamad Medical Corporation, Doha, Qatar; ^4^Psychiatry Hospital, Hamad Medical Corporation, Doha, Qatar

**Keywords:** dementia, brain injury, trauma, sport trauma, head

A recent meta-analysis reported that traumatic brain injury (TBI) increases the risk of dementia by 70%, particularly in young men and Asians (1). Another systematic review (2) showed that neuropathological diagnosis in dementia patients had been reported in 15.5% of 44 eligible studies; however, the exposure and outcome heterogeneity, in addition to the poor quality of studies, were of substantial concern. In general, the contemporary literature lacks enough data to explore the direction and interactions between dementia and trauma, including which one is a risk factor for the other and to which extent. Additionally, the link between cerebrovascular pathology, neuroinflammation, and the severity of trauma remains understudied (3, 4). To shed light on dementia associated with TBI, trauma-induced neurodegeneration and polypathology have been addressed in a few reports (5). In more than two-thirds of subjects who have dementia, at least one severe or repetitive mild traumatic event is reported before the onset of dementia.

Depression onset may occur a few years preceding dementia diagnosis, and therefore, it could be prodromal or a risk factor (increases the risk of dementia by 1.65-fold in some instances) (6). Subjects who sustained post-traumatic stress disorder (PTSD) have a 2-fold increase in the likelihood of developing dementia later in their life (in a bidirectional relationship) (7). While PTSD can follow any stressful trauma, it is more evident after spinal and head injuries. Most subjects who suffered a traumatic event will not necessarily develop PTSD, and the majority may not be asked about PTSD by healthcare professionals (8, 9). Five out of every 100 adults in the USA have PTSD in any given year (about 8/100 for females and 4/100 for males) (8, 9). Few reports estimated that dementia after PTSD ranged between 4.7% and 7.8%. The most common causes of head injury include falls (40%), motor vehicle crashes (14%) and Assaults (11%). In contrast to Western countries, the mean age of TBI patients is strikingly low in Middle Eastern countries (35–40 years), which makes tracking the development of dementia challenging in these countries, which underestimates its real incidence and association with injury.

Most TBI injuries are mild (65%), while 10% are moderate and 25% are severe. Research indicates a strong association between moderate/severe TBI and dementia, with an increased risk of dementia ranging between 2 and 4-fold; however, there is limited evidence of the association between dementia and mild TBI with loss of consciousness (LOC) (10). Individuals who sustained severe TBI-causing LOC are more liable to develop dementia (50% higher risk when compared to those without) (10). Dementia in TBI victims suggests that a risk of dementia is attributable to TBI in 5%−15% of cases (10).

Dementia-related manifestations after TBI include abnormalities of memory, thinking and concentration, communication, interactions with others, mood, and personality. TBI may cause rapid, complex structural, and physiological changes in the brain, that in addition to the released biomarkers, subsequently lead to an abrupt coping crisis and abnormal responses like excessive anxiety and depression. This disorder might happen when symptoms from psychological trauma disrupt daily functioning for at least a month. A study showed that during this time, subjects who sustained TBI were 4–6 times as likely to develop dementia than those without TBI (8, 9). Furthermore, a concussion (mild TBI) or other TBI can increase the risk of developing dementia even after 30 years of the primary insult (8, 9).

Of note, subjects above the age of 50 years old have an increase in the risk of dementia over 10 years following TBI (11). Moreover, there is a linear relationship between the risk of dementia and repeated head trauma (dose-dependent effects) (11). This observation comes mainly from sport-related cases (football and boxing) who had dementia at an older age (12). Professional football players had a 3.5-fold higher risk of death with neurodegenerative diseases (12). Moreover, there is no evidence to prove that a single, mild TBI increases the risk of dementia, but repeated mild TBI exposures can lead- to dementia in the long run.

In most cases, a diagnosis of dementia is not in itself a reason to stop driving. One in three subjects with dementia still can drive. However, over time, dementia may interfere with the skills needed for safe driving, which should be explicitly discussed with the treating physician (13). There is not enough published data on dementia-induced trauma, although it may happen with drivers who suffer significant dementia.

In countries with construction projects, falls from heights are the most common cause of TBI and the potential development of dementia in later life. Consequently, safety measures are crucial at workplaces. Also, wearing helmets for motorcycle drives, seat belts for car passengers, and implementing safety measures at home to protect elderly individuals from falls are paramount. Early referral of any TBI degree of severity following hospitalization to psychology and neurology evaluation is highly appreciated. Concussion clinics with long-term follow-up and post-injury rehabilitation psychology clinics are also necessary measures. Moreover, high-quality studies and national registries with rigorous validation are of great value in tracking the onset of dementia in high-risk patients with a history of traumatic injuries. In conclusion, despite the bidirectional relationship between TBI and dementia is a hot topic for researchers, it remains underestimated and an extensive translational research with long-term follow-up is warranted.

## Author contributions

AE-M: Conceptualization, Writing—original draft, Writing—review & editing. HA-T: Writing—review & editing. MM: Conceptualization, Writing—review & editing.
